# New Insights into Multiple Sclerosis Mechanisms: Lipids on the Track to Control Inflammation and Neurodegeneration

**DOI:** 10.3390/ijms22147319

**Published:** 2021-07-07

**Authors:** Maria Podbielska, Joan O’Keeffe, Anna Pokryszko-Dragan

**Affiliations:** 1 Department of Biochemistry & Molecular Biology, Medical University of South Carolina, Charleston, SC 29425, USA; 2 Laboratory of Microbiome Immunobiology, Ludwik Hirszfeld Institute of Immunology & Experimental Therapy, Polish Academy of Sciences, 53-114 Wroclaw, Poland; 3 Department of Analytical, Biopharmaceutical and Medical Sciences, School of Science & Computing, Galway-Mayo Institute of Technology, Galway, Ireland; joan.okeeffe@gmit.ie; 4 Department of Neurology, Wroclaw Medical University, 50-556 Wroclaw, Poland; anna.pokryszko-dragan@umed.wroc.pl

**Keywords:** central nervous system, lipids, inflammation, lipidomics, MS biomarkers, MS therapy, MS mechanisms, multiple sclerosis, neurodegeneration, neurological diseases

## Abstract

Multiple sclerosis (MS) is a central nervous system disease with complex pathogenesis, including two main processes: immune-mediated inflammatory demyelination and progressive degeneration with axonal loss. Despite recent progress in our understanding and management of MS, availability of sensitive and specific biomarkers for these both processes, as well as neuroprotective therapeutic options targeted at progressive phase of disease, are still being sought. Given their abundance in the myelin sheath, lipids are believed to play a central role in underlying immunopathogenesis in MS and seem to be a promising subject of investigation in this field. On the basis of our previous research and a review of the literature, we discuss the current understanding of lipid-related mechanisms involved in active relapse, remission, and progression of MS. These insights highlight potential usefulness of lipid markers in prediction or monitoring the course of MS, particularly in its progressive stage, still insufficiently addressed. Furthermore, they raise hope for new, effective, and stage-specific treatment options, involving lipids as targets or carriers of therapeutic agents.

## 1. Introduction

Multiple sclerosis (MS) is a chronic immune-mediated demyelinating disease of the central nervous system (CNS). The multifocal CNS injury results in MS lesion formation, designated as demyelinating plaques. Histopathological examination of CNS tissues indicates infiltration of T lymphocytes, B cells, and macrophages, as well as oligodendrocyte damage and axonal degeneration (axonopathy). MS usually exhibits multiphase course with periods of exacerbations (relapses) and improvement (remission)—typical for relapsing–remitting MS (RRMS) subtype. However, in later stages of the disease, the majority of patients present a gradual progression of neurological symptoms and disability, transforming into secondary progressive (SPMS) form. A small percentage of patients develop primary progressive (PPMS) course from the disease onset. The more recent understanding of MS disease course assumes distinguishing two main phases of the disease: active and progressive/inactive, which may be temporarily overlapping [1].

MS-related damage to the CNS is thought to be mediated by two overlapping processes: inflammatory demyelination and progressive neurodegeneration [2]. Both processes were shown to be initiated at the disease onset, but they develop with different dynamics: the peak of inflammatory activity occurs in the early stages of MS, while neurodegeneration with axonal loss is gradually escalating towards more advanced progressive stages [3]. It is also suggested that in MS patients two types of inflammation (focal and diffuse) occur, which develop in parallel but partially independent from each other [4].

**Figure 1:**
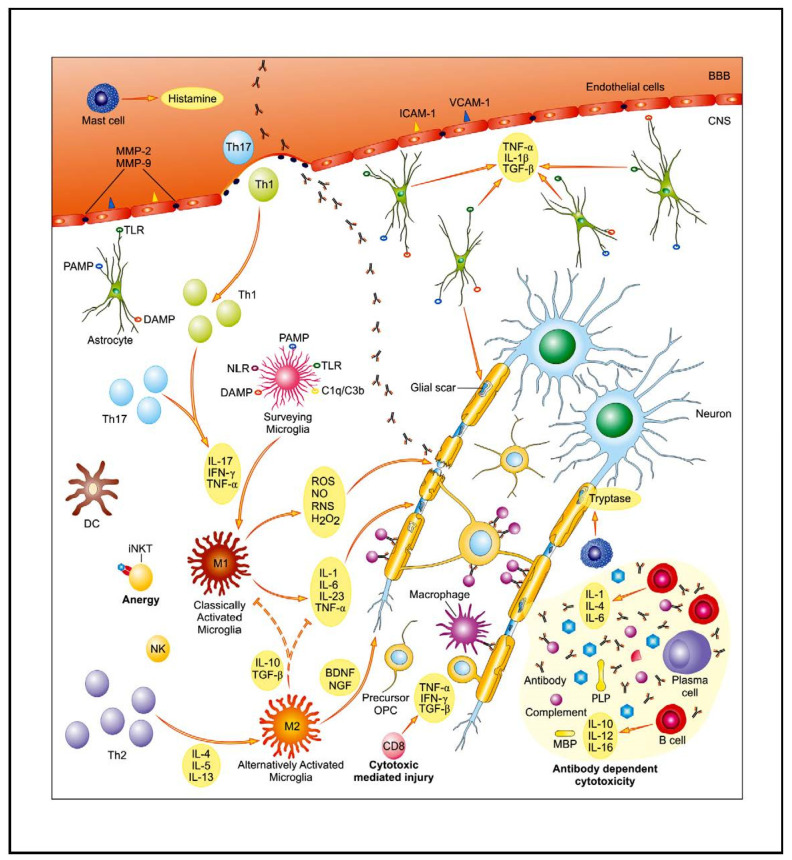
The core of multiple sclerosis (MS) background is associated with disturbed, autoreactive activity of both the innate and adaptive immunological system. As a result of complex interplay between genetic and environmental factors, pools of auto-reactive T cells are activated and enter the central nervous system (CNS) through the disrupted blood–brain barrier (BBB). Their entry is facilitated, i.e., by enhanced expression of endothelial adhesion molecules (ICAM-1, VCAM-1) and matrix metalloproteinases (MMP-2, MMP-9). An activation of glial cells further contributes to pro-inflammatory properties of the CNS environment. Multiple mechanisms of immune-mediated injury of myelin and axons have been postulated: cytokine-mediated damage, digestion of surface myelin antigens by macrophages, antibody-dependent and complement-mediated cytotoxicity, and direct cytotoxic attack by CD8^+^ T cells. Parallel to inflammatory activity, there is slowly expanding neurodegenerative injury with axonopathy. The main contributing factors include: toxic metabolites (ROS, NO, RNS), mitochondrial and peroxysomal dysfunction with energetic deficit as well as disturbed ionic balance, and emerging pro-apoptotic activity. Abbreviations: BDNF—brain-derived neurotrophic factor, DAMP—danger associated molecular pattern, DC—dendritic cell, IFN-γ—interferon γ, IL—interleukin, iNKT cells—invariant natural killer T cells, MBP—myelin basic protein, NGF—nerve growth factor, NLR—NOD-like receptors, PAMP—pathogen-associated molecular pattern, TGF-β—transforming growth factor β, Th—T helper, TLR—Toll-like receptor, TNF-α—tumor necrosis factor α. Adapted from [5].

Over the past decade, great progress in understanding the role of the immune system, both “innate immune system” and “adaptive immune system”, in MS has been made, linking them to different stages of the disease (Figure 1). Thus, while the adaptive immune system is mainly involved in the acute inflammatory events, innate immunity plays a major role in progressive phase of MS. However, the mechanisms resulting in the escalation of the autoimmune response and MS-related CNS damage are complex and have not been thus far fully elucidated. It is widely believed that MS develops in genetically susceptible individuals, with contribution of environmental factors (infectious pathogens, exposure to sunlight, vitamin D3 level, hormonal dysregulation, stress, etc.).

A great individual variability in clinical presentation in the population of MS patients, presumably determined by differences in dynamics and profile of immune-mediated underlying processes [6], makes early diagnosis of the disease, as well as prediction of its further course, still difficult. Despite thorough investigations, few sensitive and specific biomarkers have been found in this field thus far. The same refers to the possibility of monitoring of progressive inflammatory and neurodegenerative processes and predicting individual patients’ response to the applied treatment. Despite the significant progress in MS treatment, there are still some limitations and challenges ahead. Most of the available drugs modify the course of MS, without permanent cancellation of ongoing CNS damage and little potential for repair. In addition, the majority of therapeutic options targets the active phase of MS with inflammatory demyelination, while neuroprotective properties and effect upon progressive phase have been demonstrated for a few drugs only [7].

In order to obtain a better understanding of the pathophysiology of MS, we focused on the lipids that are considered to play a significant role in the disease background. Lipids are not only substantially involved in the formation of myelin sheath (and thus could be a hallmark of demyelination and repair), but are also engaged in the cell signaling, in communication, and in transport in the CNS [8]. Thus, they seem likely candidates for biomarkers of processes underlying active and progressive phase of MS, as well as potential target for new, effective, and stage-specific therapeutic interventions [9,10].

On the basis of our previous research as well as an extensive literature review, we present a current perspective of lipids as relevant players in neuroinflammation and neurodegeneration, two processes underlying MS pathophysiology.

## 2. MS Relapse

### 2.1. Th1/Th17 Lymphocyte Populations and the Cellular Immune Response

Active demyelinative lesions in the CNS, which constitute pathological substrate for clinical exacerbations, are massively infiltrated by macrophages (which contain myelin degradation products) and a multitude of lymphocytes, including CD8^+^ and CD4^+^ T cells and B cells [6]. The prevalent dogma has been that the main players of MS contributing to the inflammatory component of the pathogenesis of the disease are autoreactive CD4^+^ T helper (Th) cells, key participants of the adaptive immune response activated against a wide range of pathogens. Naïve CD4^+^ T cells are activated in peripheral lymph nodes by mature dendritic cells (DCs) that present pathogen-derived peptides associated with class II major histocompatibility complex (MHC) and together with co-stimulatory molecules promote T cell proliferation and cytokine polarization, as a consequence of which is T cell differentiation into distinct Th cell subsets [11]. This activation of lymphocytes and a failure of peripheral tolerance mechanisms results in a clonal expansion of autoreactive T cells, which subsequently enter the CNS through the disrupted blood–brain barrier (BBB) and initiate a complex cascade of inflammatory processes leading to demyelination.

It has been reported that Th1, Th17, Th9, and Th22 subsets have been associated with MS pathology, with a pivotal role of Th1 cells [12]. The activation of myelin-specific T cells accompanied by dysregulation of Th1, Th2, and Th17 secreted cytokines is considered as a major event for MS initiation [13]. An imbalance has been postulated between Th1-type pro-inflammatory response (secretion of TNF-α, IFN-γ, IL-2, IL-12, IL-15) and Th2-type response with anti-inflammatory/regulatory properties (activity of IL-4, IL-5, IL-10, IL-13) [14]. Thus, CD4^+^ T lymphocytes in MS show a clear shift towards Th1 profile response compared to healthy subjects (HS) [15]. In addition, a correlation between the severity of MS and the Th1 profile response has been demonstrated.

In more recent years, another subpopulation of Th cells (Th17), producing IL-17, was also demonstrated to play a key role in the immunopathology of MS [16]. There is an increase in IL-17 expression in both the blood and the CNS of MS patients [17] and significant increase of Th17 cells during relapses. In vitro studies have shown that Th17 cells pass through the BBB more effectively than any other T cell subsets [18]. IL-17 contributes to damage of this barrier and leads to increased infiltration of other cytokines, neutrophils, and monocytes into the CNS [19]. Moreover, by targeting resident CNS cells such as astrocytes and microglia, Th17 cells promote their activation and amplify neuroinflammation in the experimental autoimmune encephalomyelitis (EAE) [20].

In summary, all the above data signify the involvement in the effector phase of the MS pathological process of both pro-inflammatory cell populations Th1 and Th17, emphasizing the importance of their cooperation during demyelination.

#### 2.1.1. Role of Bioactive Sphingolipid Mediators in the Acute Inflammatory Demyelination in MS

Sphingolipids (SLs), the relevant components of lipid bilayers, contribute to dynamic structural and functional properties of the cellular membrane. They play an important role in organization and compartmentalization of the membrane into so-called microdomains or lipid rafts. These are involved in the processes of exo- and endocytosis, cell polarity, and intracellular signaling, as well as activation of ion channels and binding ligands to receptors [21,22]. Moreover, changes in intracellular SL levels are responsible for the dynamic balance between cell viability and destruction. Low concentrations of ceramide (Cer) were found to promote growth and division of cells, while its accumulation results in pro-apoptotic toxicity [22,23].

**Figure 2:**
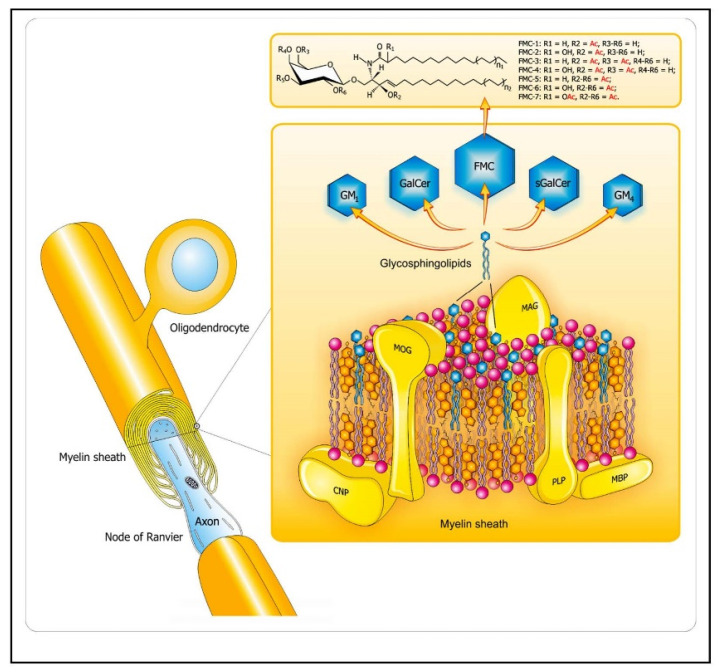
Lipid distribution in the CNS myelin sheaths. The diagram depicts arrangement of complex lipids (cholesterol, phospholipids, and glycosphingolipids) within the most abundant proteins (PLP, MBP) in CNS myelin. The relative molar constancy of lipids: cholesterol/phospholipids (PLs)/galactosylceramide (GalCer) is 2:2:1. Proteins are marked in yellow, and the comprising lipids are as follows: cholesterol in orange, PLs in pink, and the glycosphingolipids (FMC, fast migrating cerebrosides; GalCer, galactosylceramide; GM_1_, mono-sialoganglioside; GM_4_, sialosyl-galactosylceramide; sGalCer, sulfatide) in blue. Structures of unique sphingosine 3-O-acetylated-GalCer glycolipid series, namely, acetyl-cerebrosides (FMCs) are shown at the top. Abbreviations: CNP—2′3′-cyclic-nucleotide 3′-phospodiesterase, MAG—myelin-associated glycoprotein, MBP—myelin basic protein, MOG—myelin oligodendrocyte glycoprotein, PLP—proteolipid protein. Adapted from [5].

SLs are abundant lipid components in CNS, as depicted in Figure 2. Their profiles are specific for particular cell types and life stages (from early development to aging). Proper integrity and organization of neuronal membranes, provided by their lipid components, is essential for processes of neuronal polarization and differentiation, formation of synapses and interaction between neurons and glial cells, and activity and plasticity of neuronal networks. Therefore, regulation of the SL signaling network is crucial for proper function of CNS, and its disturbances were demonstrated to be involved in the background of a range of neuroinflammatory and neurodegenerative diseases [21,22,23]. SLs exert pronounced effects on inflammation in the context of autoimmunity, acting either as targets and also modulators of the immune response, with myelin sheath reported to induce apoptosis in auto-reactive T cells [24] and to ameliorate EAE [25,26].

Recent evidence suggests that alterations in the SL pathway may reflect disease activity [27,28]. In particular, Cer and the enzymes linked to its production have been described to play a pivotal role in oligodendrocyte damage and acute demyelination [26,27,29,30,31]. Qin et al. reported that, induced by staurosporine, oligodendrocyte cell death is associated with increased pro-apoptotic C16:0- and C18:0-Cer levels, increased sphingosine (Sph), and decreased sphingosine 1-phosphate (S1P) level [32]. On the other hand, SL alterations examined in vivo by Kim et al. indicate that levels of C16:0-, C18:0-, and C20:0-dihydro (dh)Cer and S1P were significantly elevated in the white matter (WM) of cuprizone-fed mice [30]. Moreover, they identified that aberrant upregulation of astroglial Cer potentiates oligodendrocyte injury [30]. Therefore, it seems that perturbations in the balance of anti- and pro-inflammatory lipids in the CNS are indeed involved in MS pathology [33,34].

**Figure 3:**
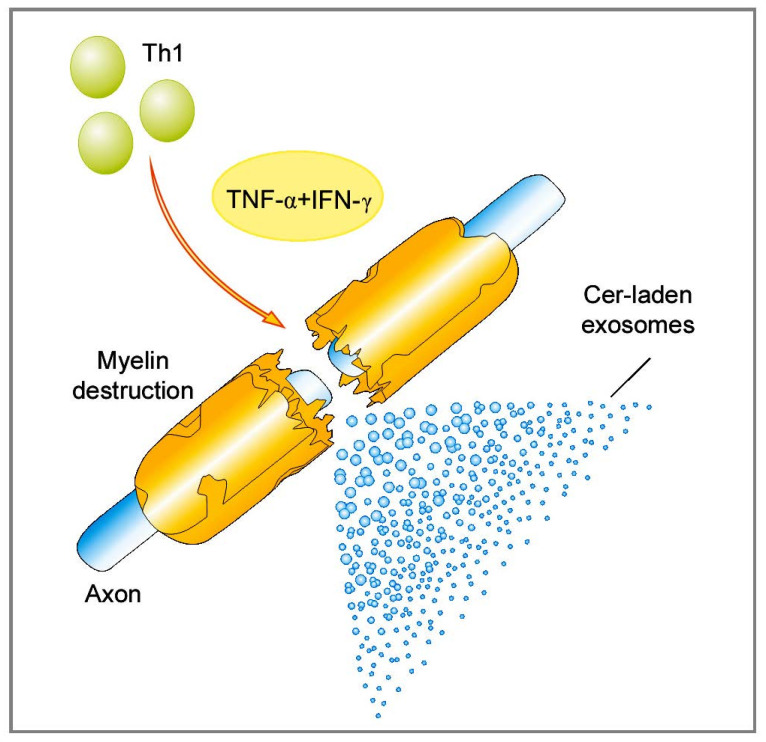
Cer-laden exosomes drive acute inflammatory demyelination in MS. Abbreviations: Cer—ceramide, IFN-γ—interferon γ, MS—multiple sclerosis, Th—T helper, TNF-α—tumor necrosis factor α. Adapted from [36].

There are also reports that highlight the involvement of Cer in a process of apoptosis induced by pro-inflammatory Th1-type cytokines [35]. Recent findings show that Cer participates in the formation of secreted microvesicles that are being increasingly recognized for their roles in intercellular communications, both under physiological and pathological conditions. We have recently explored the role of bioactive lipids, particularly Cer, in the pathomechanisms of autoimmune demyelination in MS [29]. Specifically, we examined the effect of a Th1 pro-inflammatory cytokines, TNF-α, and IFN-γ, which are known to be accumulated in MS brain lesions, upon human oligoendroglioma (HOG) cells. We have found out that upon stimulation with TNF-α and IFN-γ, the HOG cells secrete Cer-laden exosomes. Our results indicate the potential mechanism affecting oligodendrocytes susceptibility to autoimmune attack is associated with the pro-inflammatory and pro-apoptotic features of Cer. Certainly, these microvesicles offer new molecular insights into MS pathology (Figure 3). Their release from stressed or cytokine-targeted oligodendrocytes in vivo may “broadcast” the cell death signal and promote the autoimmune response that occurs under demyelinating conditions in the CNS. The investigation of exosome secretion in MS is interesting and could potentially be useful as a diagnostic or prognostic marker in the future.

#### 2.1.2. Impact of Sterols on MS Autoimmunity

Several studies suggest that cholesterol derivatives—oxysterols—may be associated with inflammatory demyelination in MS. The brain contains the highest amount of cholesterol compared to other organs, constituting about 25% of its total content [37]. About 70% of brain cholesterol is presented in myelin, while the remaining part is divided between astrocytes, microglia (20%), and neurons (10%) [38]. In the adult brain, cholesterol is produced de novo by astrocytes, followed by its transportation (by lipoprotein ApoE) to the neurons, where it is metabolized (by cholesterol 24-hydroxylase) into 24(S)-hydroksycholesterol, 24(S)-OHC. The brain is the major source of 24(S)-OHC. ApoE regulates the endogenous amount of cholesterol via nuclear receptor, namely, liver X receptor (LXR) [39]. In MS patients, 24(S)-OHC diffuses through the disrupted BBB, and in the circulation is estrified with fatty acids into either low-density lipoprotein (LDL) or high-density lipoprotein (HDL) to be transported and metabolized in acid bile in the liver. Only a small fraction of 24(S)-OHC from the brain, less than 1% of its total secretion, occurs in CSF. This oxysterol diffused into CSF indicates lipotoxic activity on the CNS cells, especially oligodendrocytes [40], and could alter cholesterol metabolism, thus affecting the myelination process. In addition, the concentrations of other cholesterol precursors: lanosterol, the first sterol formed during its biosynthesis, and the further ones—lathosterol (Kandutsch-Russell pathway) as well as desmosterol (Bloch pathway)—could be also affected by lipotoxicity induced by 24(S)-OHC.

Oxysterols are also known to influence lipid synthesis by acting on sterol regulatory element-binding proteins (SREBPs). These transcription factors regulate lipid homeostasis by activation of more than 30 genes involved in the synthesis and uptake of cholesterol, fatty acids, triglycerides, and phospholipids (PLs) [41]. Several studies highlight the importance of oxysterols produced by auto-oxidation, specifically 7-ketocholesterol (7-KC) and 7β-hydroksycholesterol (7β-OHC) in MS patients, where they appeared to be potent inducers of oligodendrocyte cell death [39]. As outlined above, these biological effects on cholesterol metabolism potentially interfere with cholesterol synthesis and compromise myelin formation [42].

Oxysterols also control the expression of free acid synthase involved in the synthesis of fatty acids. The concentrations of oxysterols, (i.e., 24(S)-OHC) passing through the BBB may modulate free fatty acid levels via LXR-mediated signaling [43].

Moreover, oxysterols may alter inflammatory events in MS. It has been shown that LXR can mediate negative regulation of mouse and human Th17 cell differentiation and polarization [44], and its agonist is capable of suppressing the production of pro-inflammatory cytokines: IL-17, IL-17A, IFN-γ, and IL-23R [45]. Due to this putative background, oxysterols were found to decrease clinical symptoms and inflammatory activity in a mouse model of EAE [44,45].

In contrast to the classical HDL-mediated reversed cholesterol transport, this sterol could be eliminated from many cells by its side chain oxidation. Almost all cells contain the enzyme sterol 27-hydroxylase to be used to convert cholesterol into 27-hydroxycholesterol (27-OHC). A positive correlation between cholesterol and 27-OHC in the circulation has been found [46]. The level of 27-OHC, outside of CNS, depends upon the integrity of the BBB, and its damage results in a higher flux of this side chain oxyterol from the circulation into the brain. In mice with acute EAE, plasma concentration of 24(S)-OHC and 27-OHC were found to be increased along with a reduced level of lathosterol [47]. Furthermore, CSF levels of 24(S)-OHC and 27-OHC were found to be increased in MS patients, altogether suggesting that the active inflammatory process could be associated with increased turnover of brain cholesterol [48].

Plasma concentration of 24(S)-OHC depends on the balance between its cerebral production—proportionally to the number of metabolically active neurons in the CNS—and hepatic elimination [49]. Over-expression of neuronal cholesterol 24-hydroxylase, increased cholesterol turnover, dysfunction of BBB, and inflammation might result in increased or reduced level of 24(S)-OHC in the plasma in particular stages of MS, constituting a suitable biomarker of the disease activity [50,51].

### 2.2. Antibody-Dependent Functions of B Cells and the Humoral Immune Response

Despite the dominant role of T cells, B cells have also been shown to display a multidirectional activity in pathomechanisms of MS [52]. B cells tend to accumulate mainly within perivascular and—to a lesser extent—parenchymal inflammatory infiltrations [6]. B cells penetrate through the disrupted BBB and transform into plasma cells, producing autoantibodies in the CNS [53]. Moreover, they can act as antigen-presenting cells (APCs), providing stimulating signals to autoreactive T cells [54], producing soluble neurotoxic components triggering demyelination [4] and switching to memory cells that contribute to auto-proliferation of CD4^+^ T cells [55].

Presence of oligoclonal bands (OCBs) of immunoglobulins G (IgG) exclusively in the cerebrospinal fluid (CSF) and brain tissue specimens (but not in serum) from MS patients is the hallmark of their intrathecal synthesis due to persistent clonal expansion of various B cell populations [56]. A separate, invariable pattern of OCBs for each patient, which is not affected by treatment, stands for a permanent dysregulation of humoral immune response. Although not strictly specific to MS, presence of OCBs in CSF and increased IgG index [57] are considered as a relevant element of MS diagnostic criteria and prognostic factors [58].

Further stages of this humoral response, including opsonization, antibody-dependent cellular cytotoxicity (ADCC), and activation complement components, consequently lead to oligodendrocyte damage in the CNS. However, whether B cells are an initiating element of MS pathogenesis or become activated secondary to Th1-mediated autoreactive response requires further clarification.

#### Molecular Mimicry of Endogenous Myelin Lipids and Pathogenic Antigens as One of the Mechanisms of Initiation of Autoimmune Demyelination in MS

Extensive, but not successful, attempts to determine the antigenic specificity of the IgG antibodies in MS patients have been undertaken. Thus far, antibodies directed against numerous target antigens: both auto- and non-self, has been demonstrated in MS patients. Initially, the research was focused on the search for Igs directed against exogenous bacterial [59,60,61,62,63] and viral antigens [64,65,66,67], according to the existing concept of an infectious nature of MS [68]. Another hypothesis was that the antibodies present in the CNS are directed against endogenous antigens derived mainly from the myelin sheath [69]. The mainstream research for an inducer of autoimmune response in MS patients has focused mainly on myelin sheath proteins [70,71,72]. Myelin lipid antigens have been neglected as candidate targets for autoimmune attack in MS for some time, although antibodies to gangliosides [73,74,75,76], sulfatides [77,78] and their complexes [79], PLs [80] as well as oxidized PLs, oxidized sterols, and sphingomyelin (SM) [79,81,82] have been found in body fluids of MS patients. Moreover, antibodies against galactosylceramide (GalCer) have been revived as potential MS immune markers [83].

The above studies suggest that humoral autoimmune response does play a substantial role in the MS pathogenesis, but no study has convincingly demonstrated particular antigen(s) as a target for MS-specific antibody response. Most antibodies detected in MS have also been found in other neurological and systemic conditions, as well as in HS. The identification of so many potential auto-antigens illustrates the complexity of the processes underlying MS pathology. Many observations, including the individual differences in the course of disease, suggest that there is no universal antigen, but rather a common mechanism of disease development, initiated by different auto-antigens in particular individuals. However, due to the overlapping presence of these biomarkers in other diseases, their specificity is still questionable, and their usefulness as potential biomarkers on disease progression and prognosis requires further research.

An attempt to identify the specificity of the antibody response was undertaken by our group while searching for specific antibodies directed against the newly discovered myelin’s lipid components, i.e., fast migrating cerebrosides (FMCs) in MS CSF [84]. It was assumed that these myelin-acetyl-cerebrosides resemble acyl- and/or acetyl-carbohydrates of lipopolysaccharide (LPS) and/or glycolipids (GLs), which can be found on the external surface of many bacteria. Considering the molecular mimicry as one of the potential pathomechanisms leading to autoimmune attack, a successful attempt to determine the structural similarities between endogenous and exogenous antigens, contributing in consequence to the cross-reactivity of antibodies directed against them, was undertaken. In these studies, the focus was on endogenous lipid antigens found in myelin sheath, including FMC-7, GalCer, and sulfatide. Exogenous antigens of bacterial origin included: (i) purified glycoglycerolipid containing phosphocholine (PC)—MfGL-II-derived from *Mycoplasma fermentans* [85] and (ii) purified LPSs isolated from *Chlamydia pneumoniae*, *Campylobacter jejuni*, *Helicobacter pylori*, and *Escherichia coli*. The observed cross-reactivity of the anti-FMC-7 antibody with the purified MfGL-II lipid and with the LPS from *E. coli J5* seemed to confirm the validity of our hypothesis. Alternatively, analysis of IgG antibodies derived from CSF obtained from patients with symptoms of neurological deficits, suggesting MS demyelinating process, classified in the main clinical subtypes and two reference groups of patients subdivided into inflammatory other neurological disease (I-ONDs) and non-inflammatory ONDs (NI-ONDs) controls was performed [84]. The I-OND group included patients with meningitis and encephalomyelitis, as well as subacute sclerosing panenecephalitis (SSPE). The highest reactivity against the most hydrophobic representative of family of acetylated β-GalCer derivatives, i.e., the FMC-7 antigen, was observed in the I-OND group. In addition, in this group, a significantly elevated level of antibodies directed against exogenous bacterial antigens derived from *M. fermentans* and *E. coli J5* was found. The studies also showed that the level of anti-FMC-7 antibodies was significantly higher in patients with PPMS compared to SPMS and in the RRMS subtype in the remission phase compared to SPMS. Only some of the MS CSF tested showed elevated antibody titers against both myelin endogenous antigens and exogenous bacterial antigens. This fact may indicate that in some MS patients, this immunoreactivity is relevant to the pathogenesis of the disease. This finding is in agreement with recognized diversity, both in pathology and clinical presentation of MS.

The results obtained by our group suggest a biologically relevant correlation between *M. fermentans* or *E. coli J5* infection, and inflammatory demyelination [84]. In the light of current research, it is believed that viral or bacterial pathogens may initiate or exacerbate autoimmune diseases through various mechanisms [68,69,86,87]. Our data illustrate the importance of molecular mimicry in the initiation of autoimmune diseases in the CNS. This pathomechanism is one of currently postulated mechanisms by which the infectious agent can potentially participate in initiating of the pathological process in MS. Other hypotheses ascribe the key role to bystander activation of the immune cells, mediated by pathogenic antigens or cross-reactivity to latent infection. The observed molecular mimicry and its connection with MS pathogenesis has been buttressed by identification of sequence homologies between myelin protein genes and viral and bacterial nucleic acids [69,88].

Taken together, however, many infectious agents have been postulated, but none have been unequivocally linked to MS yet, and there is a high likelihood that infection may play a role in initiating or causing its features. FMCs may be a potential candidate mimic targeted by immune responses initially directed at bacterial acyl- or acetyl-conjugated glycosides, which can subsequently induce autoimmune inflammation in the CNS [89]. Unlike peptides that reside internally, these complex lipophilic molecules, located on the microbial surface, most likely first encounter host immune system in the course of infection. Hence, they may qualify as possible “original antigens” igniting host immune response, including breaking tolerance; enabling autoimmune reactivity; and causing inflammation, demyelination, and symptomatic MS. This is a plausible means by which an ordinarily host-protective defensive immune response to infection (or an endogenous signal) can be transformed into self-destructive attack.

## 3. MS Remission

### 3.1. T Cells with Regulatory Features

A complex and heterogeneous picture of MS immunopathology suggests the presence of immunoregulatory abnormalities in this disease [90]. They may be the result of disturbances of functional interactions of many cellular components of the immune system, especially regulatory T cells (Treg) [91]. There are several regulatory T cell populations involved in suppression and prevention of MS development. Among these, the most important role is attributed to the thymus-derived natural Treg (nTreg) [92] with the CD4^+^CD25^+^FoxP3^+^ phenotype and Treg induced within the peripheral immune system (iTreg) [93], e.g., Tr1 and Th3 lymphocytes. The regulatory function may be also attributed to Th2 cells secreting IL-4, IL-5, and IL-13. In contrast to the pro-inflammatory Th1/Th17 cells, Th2 cells participate in modulation of inflammatory effects [94]. Current findings suggest that pro-inflammatory Th1 and Th17 cells predominate during relapse, while anti-inflammatory Th2 and nTreg drive remission [14].

In addition to the above mentioned subpopulations of CD4^+^ lymphocytes, natural killer T (NKT) cells have also been proven as immune regulators of MS [95]. NKT cells are of particular interest, because unlike conventional peptide-binding T cells [96], they recognize self or non-self lipid antigens presented by the CD1d molecule [97]. These cells constitute a heterogeneous T cell population that expresses markers characteristic of both conventional T cells such as T cell receptor (TCR) and NK cells (e.g., CD56, CD94, CD161). They can be divided into at least two subsets on the basis of their TCR. The majority of NKT cells in humans express a unique TCR encoded by invariant Vα24Jα18 α-chain gene segments co-expressed with the Vβ11 TCR β-chain (invariant TCR or iTCR); they are commonly referred to as invariant NKT (iNKT) cells or type I NKT. This population is also characterized by the ability to respond to lipids and GL antigens in the context of CD1d, the most potent being α-GalCer derived from the marine sponge *Agelas mauritianus.* NKT type II (non-iNKT) cells lack an iTCR and are more diverse. However, iNKT cells account for a small percentage of all lymphocytes, and they are extremely potent and play central roles of autoimmunity. Their action is immediate, unlike the more conventional T cell populations, and contributes to stimulating the activities of other immune system cells, including NK cells, conventional CD4^+^ or CD8^+^ T cells, DCs, and B cells. They could be activated in two ways: (i) directly, through stimulation via the iTCR by lipid antigen in the context of CD1d, and (ii) indirectly, via cytokines produced by APCs. In the direct mechanism, there are a number of lipid and GL antigens that specifically bind CD1d and subsequently prime and activate the iNKT cells to different functions that are determined by the local tissue environment, especially cytokines. The breadth of molecules reacting with the iNKT receptor defines the iTCR as an innate pattern recognition receptor (PRR) where iNKT cells bind to variety of lipid antigens complexed with CD1d. In addition to the already mentioned, the best-known, and the most effective iNKT cell activator, α-GalCer, the range of stimuli include exogenous microbial ligands and endogenous self-antigens [97].

#### Endogenous Glycolipid Ligand-Driven iNKT Cell Anergy as a Bridge of Adaptive Immune Response and Innate Immunity in MS

NKT cells perform major regulatory functions in immunity, proven in several model studies [98,99,100]; however, their function in MS is not yet fully understood. In previous studies, our group demonstrated numeric changes in circulating peripheral blood iNKT cells in MS [101]. To determine the functional significance of these ex vivo quantitative changes, we also performed further functional studies. We used the myelin-derived lipids, namely, a mixture of FMC-5 and FMC-7 or purified FMC-7 [84] as ligands to stimulate iNKT cells from patients with MS compared with HS controls in vitro. These cells failed to respond to in vitro stimulation in the MS cohort, suggesting a form of anergy that was in marked contrast to the broad range of Th1, Th2, and Th17 cell cytokines produced by HS controls [102]. The results showed that both FMC-5 and FMC-7 mixture and purified FMC-7, in addition to α-GalCer, the strongest activator of iNKT cells, were potent activators in HS controls, and importantly the response was specific to iNKT cells (CD3^+^ Vα24Jα18^+^). The purified FMC-7 induced the proliferation and secretion of Th1-type cytokines: TNF-α, IFN-γ, IL-1β; Th2 type: IL-6, IL-10; and Th17 type: IL-17 in the HS control group. In turn, α-GalCer preferentially induced the production of Th1 type pro-inflammatory cytokines, including TNF-α, IFN-γ, and IL-2, in addition to the only observed Th2 type cytokine, i.e., IL-6. These were novel findings, wherein a myelin-derived lipid induced activation of peripheral blood iNKT cells [102]. Our molecular model suggested that the conformation of the poly-acetylated galactose residue in FMC-7 matches the binding site of the iTCR receptor, resulting in the initiation of iNKT cell activation. This is in line with the current concept of the “glycolpid moulding” in the CD1-GL-iTCR synapse. The reason for the observed hyporesposiveness of iNKT cells [102] remains to be clarified in the MS subjects but may possibly be due to earlier contact with GL or GSL antigens in vivo, resulting in saturation of the iTCR receptor with a GL bacterial ligand during infection or GSLs released from myelin during the demyelinating process.

**Figure 4:**
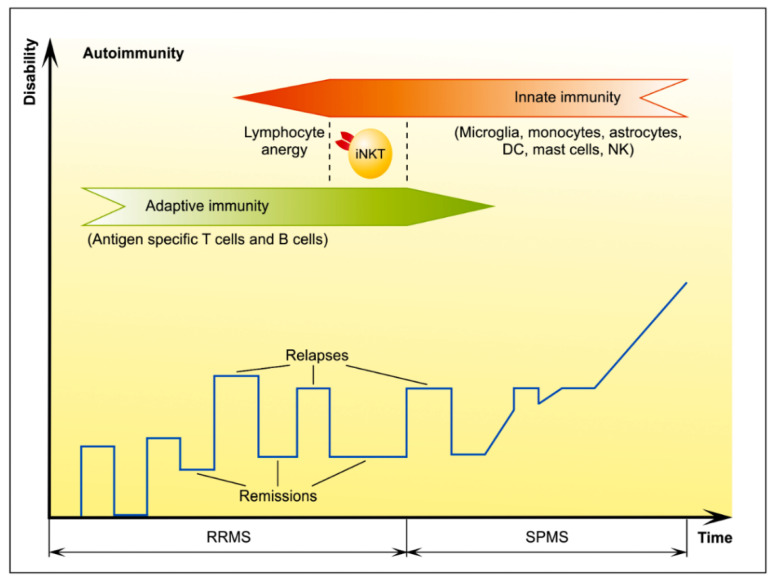
The iNKT cells act as a bridge of adaptive and innate immunity. A clinical hallmark of MS is the heterogeneous presentation ranging from benign with little or no disability even years after disease onset, a commonly encountered a relapsing-remitting (RR) phase followed by a secondary progressive (SP) phase, progressively disabling course. The RR phase is driven by the adaptive immune response while SP phase is driven by the innate immune system. We propose invariant natural killer T (iNKT) cells act as a bridge between adaptive and innate immunity, the phase characterized by lymphocyte anergy resulting in MS remission. Abbreviations: DC—dendritic cell, NK—natural killer, iNKT cells—invariant natural killer T cells, RRMS—relapsing–remitting multiple sclerosis, SPMS—secondary progressive multiple sclerosis. Adapted and modified from [5].

Rendering iNKT-cells hyporesponsive to endogenous GSLs is a novel insight into diseases manifesting aberrant iNKT-cell activation, and consequently this finding of GL ligand-driven anergy in MS has substantial implications [89]. Furthermore, the state of anergy following stimulation with the auto-antigen FMC-7 may have significant clinical implications, according to the theory of antigen-specific therapy of autoimmune diseases. Diverse GLs including the endogenous myelin acetylated-β-GalCer, FMCs, can drive activation critical to controlling CNS inflammation and fostering myelin repair. Since α-GalCer reduces the symptoms of the disease, as demonstrated in vivo on the EAE model [103], and supports remyelination, the role of FMCs in a similar mechanism in MS seems highly likely. It was also reported that stimulation of T lymphocytes with sulfatide in vitro led to inhibition of Th17 cell differentiation and proliferation, as well as reduction of clinical symptoms of the disease [104]. A better understanding of the molecular basis for the observed anergic response of iNKT cells during CNS autoimmunity and the contribution of other cells and cytokines in the mechanisms underlying the impaired immune response in MS will help to develop improved treatment strategies in the future. However, we propose iNKT cells anergy as a bridge between adaptive and innate immunity, a phase resulting in MS remission (Figure 4).

## 4. MS Progression

### 4.1. MS Neurodegeneration Background

During the progressive phase of MS, neurodegenerative processes with axonal and neuronal loss predominate over inflammatory demyelination, and thus accumulating damage to the CNS overweighs its compensation from repair and functional reserve. It is currently believed that this phase of MS is associated with over-activated microglia [105]. Microglia contribution seems to be very important because of their release of pro-inflammatory cytokines [106,107] and proteolytic enzymes [108,109], as well as the generation of reactive oxygen species (ROS) [110] and nitric oxide (NO) [111], particularly responsible for oxidative stress. These processes are further enhanced by mitochondrial dysfunction due to reduced neuronal expression of mitochondrial genes and accumulating mitochondrial DNA mutations. Structural and functional mitochondrial alterations include modifications in components of mitochondrial membrane, with emerging loss of transmembrane potential, inhibition of succinate dehydrogenase activity, enhanced production of O_2_^●−^, and increase in the number and size of mitochondria, probably compensatory for their dysfunction [112,113].

In addition, peroxysomal dysfunction is responsible for further disequilibrium of the redox status, with inappropriate detoxification of ROS and dysregulation of protein and fatty acid oxidation [112]. Consequently, increased energy demand of demyelinated neurons cannot be compensated and results in their damage.

Apart from energy deficit, other presumable mechanisms of neuronal loss include a disturbed ion homeostasis [114]. It is of note that under pathological conditions, changes in the expression and function of Na^+^–Ca^2+^ exchangers (NCXs), expressed in CNS cells, have been associated with neurodegeneration. Apart of Na^+^ and Ca^2+^, it has been found that activity of these NCXs is regulated allosterically by non-transported ion species, phosphatidyl inositol biphosphate, and another acidic PLs, as well as an interacting fatty acid-binding protein referred to as soluble cytosolic regulatory protein or regulatory protein of the squid nerve sodium calcium exchanger [115,116,117], and their appreciation is growing. Reverse mode of the NCXs activity has been proposed to trigger Ca^2+^ overload, thereby compromising mitochondrial function and oligodendrocyte viability [118]. An increased influx of Ca^2+^ into axons is followed by activation of proteolytic enzymes and further retrograde degeneration due to multifocal axonal injury [119,120,121]. A close functional relationship between neurons and microglial cells, the long-lived and self-propelling microglia response to neuronal cells damage, leads to “reactive microgliosis” [122,123].

Progressive phase of the disease is reflected mainly by the presence of chronic inactive MS lesions with few active chronic ones; the most typical findings at this stage include mixed active/inactive chronic MS plaques and slowly expanding (smoldering) lesions [6]. Chronic inactive MS (In-MS) lesions are characterized by a demyelinated center, which contains persistent axonal transections, while smoldering lesions show a thin rim of activated microglia with only a few myelin-containing macrophages [124]. In mixed active/inactive lesions, the central part is surrounded by a dense rim of microglia and macrophages containing myelin degradation products, T cells, and axonal transections, with ongoing demyelination at the lesion edges [124,125].

The lesions are located within white matter, but also*—*with increasing number*—*within the cortex and subcortical gray matter. An extensive damage to cortex in progressive MS is associated with subpial aggregates of B cells and T cells resembling tertiary lymph follicles, postulated to produce soluble pro-inflammatory components [126]. Abundance with cortical lesions also correlates well with microglial activation [127].

Apart from focal chronic inflammation compartmentalized within CNS, proved by appearance of demyelinative lesions, pathological findings typical for progressive MS include a diffuse white and gray matter injury. Its inflammatory component is represented by infiltrates located in perivascular spaces and diffused into the CNS, while the neurodegenerative component is characterized by axonal degeneration and reactive astrogliosis [6].

#### 4.1.1. Impact of Lipids on MS Progression

Recent studies suggest that lipid signatures can reveal potential biomarkers associated with disease progression, as well as provide novel information about pathomechanisms involved in progressive MS. Targeted sphingolipidomics have been employed to establish comprehensive SL profiles in chronic MS lesions [128]. Interestingly, our data on ex vivo SL analysis of postmortem tissues obtained from individuals with clinically diagnosed MS indicated distinctive lipid profiles in chronic MS lesions. Chronic active MS (Ac-MS) lesions were characterized by a significant increase of major dhCer subspecies in comparison to normal appearing white matter (NAWM) of normal CNS (nCNS). Contrarily, In-MS lesions were characterized by decreased level of dhCer, Cer, and SM subspecies, whereas level of hexosylceramide (HexCer) and ceramide-1-phosphate (C1P) subspecies was significantly increased in comparison to NAWM of nCNS, as well as Ac-MS plaques. Thus, we have identified a key “pathological switch”, where Cer source and its metabolic transformations turn on to result in either Ac-MS or In-MS lesions. It is widely known that Cer plays a central role in SL metabolism. The extraordinarily complex regulation of its intracellular level ranges from de novo biosynthesis, through re-acylation of sphingosine (salvage pathway) and SM hydrolysis, to GSLs breakdown [129]. The elevated level of Cer may result from impaired expression of dhCer desaturase or sphingomyelinase. Our data implicated de novo SLs biosynthesis in Ac-MS lesions. A different pathological scenario was noticed for inactive MS CNS damage, where SM→Cer→HexCer metabolic pathway could be responsible for the observed effect of damage to neurons, which is in agreement with the previous report [130]. The most important observation, related to our discovery, is striking increase of the C1P levels in In-MS lesions. We proposed C1P to be a potential new biomarker of MS progressive phase. We speculated that C1P capability to mediate arachidonic acid release [131] and also to activate group IVA cytosolic phospholipase A_2_α (cPLA_2_α), the rate limiting releaser of arachidonic acid used for production of pro-inflammatory eicosanoids [132], can diminish arachidonic acid release and downstream generation by eicosanoid producers such as cyclooxygenase (COX)-1 or COX-2 [133].

Wheeler et al. analyzed the SL content of an altered gray matter (GM) and WM in Ac-MS and In-MS in great detail [27]. SL decreased only in the altered GM in Ac-MS compared to GM of nCNS, which were identified to be C18:0- and C20:0-Cer as well as C20:0- and C22:0-SM. SL that were decreased in the altered GM of Ac- and In-MS in relation to nCNS were established to be C20:0-Cer and C22:0-Cer and C16:0-SM. Contrary to GM, a quantitative analysis of SL in the altered WM showed that only a small number of SL were decreased in Ac- or In-MS; however, all SL decreased in Ac-MS were also decreased in In-MS. SL decreased in the altered WM of both Ac- and In-MS in comparison to NAWM of nCNS, were found to be C22:0- and C24:0-SM, as well as C18:0- and C20:0-Cer.

Further understanding of the impact of lipid alterations on MS progression was provided by Pousinis et al. [8]. They developed untargeted lipidomic of postmortem WM tissues in order to differentiate potential lipid-based biomarkers between PPMS and SPMS disease subtypes as well as between both PPMS and SPMS compared to samples from cerebral NAWM. A total of 44 lipids were identified as significant lipid markers of progression in PPMS compared to SPMS, whereas a group of 10 lipid markers differentiated progressive forms of MS from NAWM of nCNS. Different panels of lipids that belong to classes of PLs, SLs, and glycerophospholipids were established to differentiate both separations, although PLs were the dominant lipids class for both comparisons. The lipids that were decreased in SPMS compared to PPMS included phosphatidylethanolamines (PE), putative PL-plasmalogens (PC-P, PE-P), phosphatidylanisols (PA), phosphatidylinositols (PI), and phosphatidylserines (PS), whereas C1P, diacyloglycerols (DG), PC, phosphatidylglycerol (PG), and lysoPLs (lysoPE, lysoPC) were increased in SPMS compared to PPMS. The lipids attenuated in PPMS and SPMS, compared to NAWM of nCNS, were established to be PA, PE, PI, PS, DG, and sulfatide. Moreover, lipids that were found to be increased in PPMS and SPMS compared to NAWM of nCNS were identified as C18:2/C20:0- and C20:4/C20:0-PE. Changes in these lipids reflected different biochemical pathways, mainly glycerophospholipids, glycosylphosphatidylinositol (GPI)-anchor biosynthesis, linoleic acid, and alpha-linoleic acid metabolism. These findings are consistent with the previous ones that reported altered levels of glycerolipids in plasma of PPMS patients [134] as well as glycerolipids and PLs in CSF of MS patients [135,136].

Amatruda et al. employed lipidomic analysis of plasma of PPMS to identify lipids related with faster clinical deterioration [137]. They found a significantly increased level of C20:0-HexCer and decreased levels of C14:0-SM and C18:2-lysophospatidic acid (LPA) in those with rapid progression. The authors concluded that the reduced level of C20:0-HexCer level in the plasma may depict more sensitively the ongoing myelin and cellular damage affecting the CNS, whereas decreased level of C14:0-SM reflects diminished neuroprotective effect mediated through signaling pathways. In addition, bioactive lipid, LPA, plays multifunctional role in the CNS. LPA-mediated signaling can influence a variety of neural processes including but not limited to oligodendrocyte maturation, neuronal plasticity, and synaptic connections [138]. Thus, decreased levels of C18:2-LPA in the plasma of PPMS patients with faster progression may be related to neurodegeneration and decline homeostasis of oligodendrocytes. The high levels of C18:2-LPA in those with a milder progression may be attributed to a compensatory mechanism and preserve neurons.

Experimental studies over the past decade have clearly shown that changes in SL metabolism commonly underlie pathological processes of neurodegeneration and inflammation, thereby suggesting that the multiple targets/steps identified in the complex network of lipids are worth detailed study. Recent evidence for the toxic role of Cer per se in the autoimmune demyelination found in MS seemed to support this hypothesis. Cer accumulation in MS plaques were reported with increases in C16:0- and C18:0-Cer [32], as well as a significant elevation of C18-Cer and C18:1-, C24-, and C24:1-Cer, showing a trend towards an increase [30]. Moreover Qin et al. reported that neurons recycle S1P to Cer, which results in increased pro-apoptotic C16:0- and C18:0-Cer levels and have consequences for their apoptosis [32]. Moreover, of relevance to the potential role of Cer in MS neurodegeneration, a recent report showed that C16:0- and C24:0-Cer as well as C16:0-HexCer were enriched in MS CSF at sufficient concentrations to induce mitochondrial dysfunction and axonal damage [130]. Furthermore Mayo et al. detected increased lactosylceramide (LacCer) levels in the CNS during the progressive phase of EAE mice [25]. They also found high LacCer levels in Ac-MS lesions, suggesting that high B4GALT6 activity and LacCer levels were also linked to MS pathology.

The “inside-out” signaling of S1P is also important platform of studies related with neurodegeneration in MS. S1P receptors (S1PRs) are expressed in a variety of cell types including immune and CNS cells, and are thought to play an important role in MS indicating a possible link between S1P signaling and neurodegenerative arm of the disease. It should be emphasized that four of the five S1P receptors (S1PR1, S1PR2, S1PR3, S1PR5) are expressed in both cell types—astrocytes and oligodendrocytes [139]—and increased levels of S1PR1 and S1PR3 have also been reported in MS lesions [30,140,141]. In addition, studies have shown that S1P signaling is a critical factor in astrogliosis. Moreover, modulation of S1PRs suppresses pathogenic activities of astrocytes in murine and human models and ameliorates disease pathogenesis in an experimental model of SPMS [142].

It was also shown that S1P induces apoptosis in neurons. Hagen et al. indicated that caspase-dependent apoptosis in lyase-deficient neurons could be induced only when these cells were incubated with S1P (by unknown mechanism followed by de-phosphorylation and re-phosphorylation by Sph kinase 2 (SphK2)) but not Sph [143].

Increased S1P concentration in MS CSF implicates bioactive lipid mediators in the process of neurodegenerative arm of MS development, and a correlation between S1P levels in CSF and in blood indicates that S1P does not simply partition passively into CSF. The increase of CSF S1P concentration may not directly reflect neurological dysfunction in MS and likely reflects transitory S1P increase during the development of MS plaque in CNS parenchyma and mediates cellular effects [144]. Previous observations in rodents showing that S1P displays strong pro-inflammatory activity, and striatal astrocytes from mouse embryos subjected to S1P undergo gliosis may predict a pathophysiological phenotype found in some MS lesions [145]. Further, it has been observed that the conditional S1PR1 knockout in astrocytes attenuates astrogliosis and EAE consistent with the notion that hyper-activation of astrocytes occurs via increased S1P release [146].

Oxysterols constitute another group of lipids of potential relevance for MS progression studies. They might appear as markers of mitochondrial status as well as neuronal damage [50]. As mentioned above, mitochondrial dysfunction is one of the key factors in neurodegenerative process underlying MS progression. It contributes to increase oxidative stress levels, i.e., by overproduction of ROS, which further promotes formation of oxidized lipids [147]. Several studies suggest that oxidative stress is associated with increased levels of oxysterols produced by auto-oxidation (7-KC, 7β-OHC) as well as by enzymatic reaction, 24(S)-OHC [48]; all of which are indicators of neurodegeneration in MS [50]. It was also reported that 24(S)-OHC, the marker reflecting the number of metabolically active neurons [50], can disrupt red-ox homeostasis of neurons and glial cells, favoring accumulation and peroxidation of lipids [148]. It is of note that cholesterol 24-hydroxylase promoter activity was induced by oxidative stress and a dexamethasome/IL-6 combined treatment [149]. The expression of cholesterol 24-hydroxylase by non-neuronal cells (astrocytes, microglia, macrophages) and impairment of the BBB function could further increase the level of 24(S)-OHC in the brain [49]. Thus, oxysterols seem to be a relevant biological marker for major processes underlying MS progression.

The human brain is highly susceptible to oxidative stress due to elevated content of easily peroxidable fatty acids, such as polyunsaturated fatty acids (PUFA) [150]. In MS patients, a higher level of the oxidative stress biomarkers: total hydroxy-octadecanoic acid (HODE), 9-HODE, and 12-HODE, was observed [151]. The increased level of fatty acid degradation products as well as cholesterol auto-oxidation products (7-KC, 7β-OHC) could induce neuronal apoptosis, contributing further to chronic injury of CNS tissues [148]. Lipid peroxidation products, such as malondialdehyde (MDA), 4-hydroxy-2-nonenal (4-HNE), 4-hydroxy-2-hexenal, and isoprostanes, cross-react with proteins and enhance oxidative stress conditions [150]. The 4-HNE, present in elevated concentrations in foamy macrophages and astrocytes in active demyelinating MS lesions [150], along with ROS, were found to be detrimental to CNS cell viability, as well as BBB integrity and functionality [152]. Several studies have indicated that increased levels of ROS and lipid peroxidation products in the CSF and plasma of MS patients, along with mitochondrial damage, strongly reiterates the importance of oxidative damage in MS progression [112,147,150,153,154].

#### 4.1.2. Lipids as Potential Biomarkers of the MS Course

Functional analysis of the lipid alterations described above (Table 1) is required to fully understand mechanisms underpinning MS progression where some relevant relationships have been observed between lipid components and various indicators of disease activity as well as its progression.

Positive correlations for C16:0-HexCer and C24:1-HexCer [155] as well as negative correlation for 16:0-lysoPI [135] in MS CSF and a degree of disability in the Expanded Disability Status Scale (EDSS) were noted. Furthermore, altered level of lysoPC in MS CSF was associated with altered plasma levels of lysoPC, lysoPE, hydrocortisone, and oxidized fatty acids; the changes related to rate of relapses or increase in disability over time [159]. Plasma levels of C20:0-HexCer were increased, while C18:2-LPA decreased in the subjects with progressive MS, with the latter showing inverse correlation with rate of deterioration in neurological deficit [137]. In RRMS patients, a smaller increase in plasma level of HDL cholesterol (HDL-C) during 5 years of follow-up was observed for those who converted to SPMS within this period [158]. Additionally, a significantly increased level of C18:0- and C18:1-lysoPC was reported in the CSF of MS patients, which correlated to the Link index, a marker of intrathecal synthesis of Igs [135].

Some relationships were also observed for radiological measures of the disease course. Serum levels of 24(S)-OHC negatively correlated to normalized brain volume measurements in magnetic resonance image (MRI) of RRMS patients [157]. Levels of C20:0-HexCer showed correlation with indices of global brain atrophy at the one-year follow-up in patients with primary progressive type of MS [137]. During 5 years of follow-up, a positive correlation was observed between increase in LDL cholesterol (LDL-C) plasma levels and occurrence of new T2 lesions, while increase in HDL-C inversely correlated with rate of cerebral gray matter atrophy in MRI, both for relapsing–remitting and progressive MS [158]. Moreover, this association remained valid after adjusting for serum level of neurofilament light chains (NFL), considered as a non-specific but sensitive marker of neurodegeneration [160].

These findings indicate lipids may be relevant markers, potentially useful in prediction or monitoring the course of MS, particularly in the progressive types of disease, for which evaluation has not yet been sufficiently addressed. Furthermore, this intricate scenario offers potential for new therapeutic targets.

## 5. Current and Future Lipids-Based Therapeutical Implications

Elaborated on the basis of lipid-associated investigations, modulators of S1PR constitute a representative group among disease modifying therapies (DMTs) currently being used in MS. The first agent approved for treatment of active RRMS, fingolimod, is a structural analogue of sphingosine, phosphorylated in vivo to its biologically active form. Fingolimod binds to four out of the five known S1PRs to produce their prolonged down-regulation [161]. Its main mode of action is associated with limited migration of lymphocytes with their sequestration in the lymph nodes. Besides prevention of autoreactive T cells entering CNS, the drug also affects the development and function of T cell subsets and contributes to their apoptosis. Therapeutic response to fingolimod in active RRMS (especially beneficial in rapidly evolving type of disease) includes reduction in relapse rate and accumulation of relapse-related disability, a decrease in new lesions in CNS shown in MRI, and a decreased rate of brain atrophy. Evidence from clinical trials and further observation has encouraged the preparation of next generation of S1P modulators [162,163,164].

Ozanimod is an agonist of the S1PR1 and S1PR5. Its effects contribute to S1PR1 internalization and degradation, preventing its reinstallation in the cell membrane. Despite the much lower affinity compared to S1PR5, by binding to this receptor, ozanimod can activate specific cells in the CNS, promote myelin regeneration, and prevent neuronal loss. Ponesimod, an iminothiazolidinone derivative, has a potent effect mainly on S1PR1, resulting in its internalization, degradation, and functional antagonism. Both drugs have been approved for use in active RRMS, with beneficial effects demonstrated for clinical and radiological measures of the disease activity [163,164].

Siponimod, a selective modulator of S1PR1 and S1PR5, is the only agent approved for treatment of SPMS. It has been shown to reduce the risk of confirmed disability progression as well as increase of new lesions load and cerebral atrophy in MRI [165]. Siponimod influences both peripheral B and T cells, penetrates through the BBB, and interacts with CNS tissues. There is evidence from preclinical studies that siponimod exerts neuroprotective effects, preventing a loss of neurons and synaptic degeneration, modulating the activity of microglia, and ameliorating the degeneration of oligodendrocytes, as well as having some potential to promote remyelination [162].

There are ongoing clinical trials with amiselimod, which was designed as a highly selective S1PR1 functional antagonist without S1PR3 activity, in order to avoid or minimize cardiological adverse effects (bradycardia, conduction blocks) [162].

Dimethylfumarate (DMF) is another disease-modifying medication, widely and effectively used in RRMS. Its immunomodulatory properties are associated with decreased numbers of lymphocytes and a shift in Th subpopulation (from Th1 and Th17 and towards Th2), resulting in reduced inflammatory infiltration and production of cytokines. Furthermore, DMF exhibits anti-oxidative activity, mainly mediated by activation of nuclear factor (erythroid-derived 2-like 2 (Nrf2)) and other transcription factors [166]. Experimental studies on murine oligodendrocytes [154] demonstrated that DMF (and its main metabolite) prevents oxidative stress by counteracting cytotoxicity induced by 7β-OHC. These effects included upregulation of anti-oxidative mechanisms, maintaining membrane integrity and reducing lipid peroxidation, as well as diminishing mitochondrial and peroxisomal alterations. Attenuating 7β-OHC-induced oxidative injury, DMF also prevented cell death due to apoptosis and autophagy, which indicates both anti-inflammatory and neuroprotective potential of this drug. In addition, DMF showed ability to normalize fatty acid profile and level of PLs, relevant for myelin integrity [154].

Similar observations on animal oligodendrocytes model were made for biotin [112], which was also counteracting 7β-OHC cytotoxic effect. Biotin was shown to stabilize and enhance antioxidant activities, as well as to restore impaired mitochondrial function. It regulated biogenesis and metabolism of lipids, especially cardiolipins and saturated fatty acids, favoring those with anti-inflammatory and anti-oxidative properties. The potential to normalize energetic processes and promote myelin formation encouraged several therapeutic trials where high doses of biotin were administered mainly in progressive types of MS. However, no sufficient evidence was obtained for beneficial effect of biotin on disease progression in these studies [167].

Another drug modulating SL metabolism is Miglustat, a GluCer synthase inhibitor, currently used in lysosomal storage disorders (Gaucher and Niemann–Pick disease). Miglustat was found to suppress chronic progressive EAE, mainly ameliorating enhanced astrocytes activity. Thus, it seems a good candidate for therapeutic use in progressive MS [168,169].

Perturbed pathways of PUFA metabolism (mostly alpha-linolenic and linolenic acid—omega-3 and -6) were postulated as another potential therapeutic target in MS. Oral administration of resolvin D1, a metabolite of the ALA pathway, to B6 mice induced with chronic EAE, ameliorated course of disease. Potential mode of action included inhibition of inflammatory responses (activity of T cells, macrophages, and microglia) and upregulation of Treg [170]. The effects of omega-3- and omega-6-derived lipid mediators—resolvin, lipoxin, and neuroprotectin—were also investigated upon human leukocytes and brain endothelial cell line. A reduction in the inflammatory response was demonstrated, with decreased activation and migration of monocytes, inhibiting endothelial dysfunction [171].

Changes of the lipid mediator profile and inhibition of 5-lipooxygenase (i.e., expressed in cerebral demyelinative lesions) may be induced by boswellic acids, contained in herbal extracts. Clinical phase II trials in RRMS during 8 months’ treatment with standardized frankincense extract showed reduction in levels of lipid mediators derived from 12 and 5-LO pathway in plasma, correlating with MRI measures and level of serum NFL [172].

Activation of LXR was found to stimulate and modulate myelin gene expression on many levels, as well as to promote oligodendroglial cell maturation and their remyelinating activity. Thus oxysterols, acting as LXR agonists, seem to be promising candidates for remyelination-inducing therapies. Indeed, some remyelination—beside suppressed chronic inflammatory injury—was seen after their administration in an animal model of EAE [50].

### 5.1. Monitoring Therapeutic Response

Lipid metabolites are currently believed to be useful biomarkers because of their dynamic profile changes in various conditions. The lipidomic approach may be beneficial in examining the effects of therapeutical interventions and has been suggested for use in monitoring in clinical therapeutic trials [10]. A few recently published studies explored this approach with regard to MS patients treated with DMT.

In the patients with RRMS, treated with DMF, significant changes were shown in plasma lipid profile after 6 months of treatment, in comparison with the treatment-naïve controls. The findings included an increase in PLs, lysoPLs, and plasmalogens, along with a decrease in circulating free fatty acids. Correlation was noted between altered levels of fatty acids and lymphocyte count (CD8^+^ T cells) in the patients [173]. Treatment with natalizumab (NZ) in patients with active RRMS resulted in reduction in CSF concentration of 24(S)-OHC and 27-OHC, which was supposed to indicate improved integrity of BBB and diminished neurodegeneration [156].

Lipid profiles were analyzed and monitored in a heterogeneous group of RRMS patients receiving various DMTs [174]. In those treated with IFN-β, increased plasma levels of Cer with particular chain length were observed in comparison with the evaluation performed prior to treatment. Treatment with NZ was associated with increase of S1P and sphinganine-1-phosphate. No alterations in lipid profiles were found during treatment with fingolimod. However, no significant correlations were shown between SL levels and clinical or other disease-related measures.

Considering potential cardiological adverse effects of S1PR modulators, basic lipid profile was assessed in fingolimod-treated patients [175]. During 12 months of follow-up, there was significant but modest elevation in total cholesterol and HDL, while LDL and triglycerides remained unchanged. The authors suggested routine monitoring of lipid profile only in the patients with pre-existing cardiovascular comorbidities.

Therefore, lipids might be considered as markers of therapeutic response and side effects of DMT, helpful in identification or prediction of non-responders and non-adherents to particular therapies.

### 5.2. Targeting CNS with Therapeutic Agents

Extracellular vesicles (EV) are nanosized particles, transporting a range of active molecules and thus playing a substantial role in intercellular communication and processes of homeostasis. Nanocarriers allow for more efficient transport of molecules to the CNS through the BBB and their selective delivery to specific targets. Lipid-based nanocarriers have many advantages over other EV, including better biocompatibility, stability, biodegradability, and lower cytotoxicity, as well as greater tendency to cross the BBB and potential to encapsulate both lipophilic and hydrophilic molecules. These properties encourage their use in novel therapeutic options [176,177].

Lipid-based EV have been tested on animal models of MS and were found to be effective in delivering DMF, teriflunomide, and corticosteroids directly to the CNS. EV enriched with particular lipids—S1P, GalCer, and sulfatides—were shown to stimulate oligodendrocytes and to promote remyelination, promoting repair of MS-related CNS damage. Overall, EV may represent a promising therapeutic strategy to modulate processes of neuroinflammation and neurodegeneration underlying MS [176,177].

## 6. Conclusions and Future Directions

MS is undoubtedly a complex disease in many aspects. Although the two main processes involved in MS background—inflammatory demyelination and neurodegeneration—have been identified, the nature of their initiation and interrelations has not been fully elucidated. There is a variety of immunocompetent cell types and biochemical mediators engaged in MS pathophysiology at particular stages of the disease, which results in highly variable and unpredictable clinical course. Despite recent progress in understanding and management of MS, there are still many challenges ahead. These include recognizing interactions between immune-mediated inflammation and neurodegeneration; the search for sensitive and specific biomarkers for these both processes; and developing new therapeutic options with neuroprotective potential, targeted at the progressive phase of disease.

Recent studies suggest that alterations of lipid metabolic pathways may play a key role in MS patomechanisms. SL, PL, glycerolipid, and sterol subspecies appear to be promising subjects of investigation in this field. Lipid-related mechanisms discussed in the above review are relevant for active inflammatory demyelination, affecting antigen recognition, as well as the activation and regulatory function of iNKT and T cells. Other metabolic pathway alterations contribute to chronic inflammatory injury and oxidative stress underlying neurodegeneration. These insights highlight potential usefulness of lipid molecules as biomarkers in the prediction or monitoring of the disease course of MS, particularly in its progressive stage, still insufficiently addressed. Furthermore, the reviewed research data raise hope for new, effective, and stage-specific treatment options, involving lipids as targets or carriers of therapeutic agents. These putative diagnostic and therapeutic implications of the novel findings encourage further development of the lipid-based investigations.

## Figures and Tables

**Table 1:** Potential lipid markers in MS.

Proposed Lipid Subspecies	Altered in/Compared to	Material	Reference
C18:0-dhCer↑, C18:1-dhCer↑, C24-dhCer↑, C24:1-dhCer↑	Ac-MS/nCNS and Ac-MS/In-MS	Plaque	[128]
C18:0-SM↓, C18:1-SM↓, C24-SM↓, C24:1-SM↓	In-MS/nCNS and In-MS/Ac-MS	Plaque	[128]
C16:0-HexCer↑, C18:0-HexCer↑, C18:1-HexCer↑, C24:0-HexCer↑, C24:1-HexCer↑	In-MS/nCNS and In-MS/Ac-MS	Plaque	[128]
C16:0-C1P↑, C18:0-C1P↑, C18:1-C1P↑, C24:0-C1P↑, C24:1-C1P↑	In-MS/nCNS and In-MS/Ac-MS	Plaque	[128]
Total LacCer↑	MS/WM of MS	Plaque	[25]
C18:0-Cer↑	Ac-MS/nCNS	Plaque	[30]
Total Cer↓, Sph↑, S1P↓, C16:0/C24:0-Cer ratio↑, C18:0/C24:0-Cer ratio↑	MS/nCNS, WM of AD	Plaque	[32]
C26:1-C1P↑, C18:0/C15:0-DG↑, C18:2/C19:0-DG↑, C10:0-lysoPC↑, C17:0-lysoPC↓, C22:2-lysoPE↑, C18:1/C18:0-PA↑, C18:1/C21:0-PA↓, C20:4/C20:0-PA↑, C21:2/C24:0-PA↓, C22:6/C18:1-PA↓, C20:5/C18:2-PC↑, C18:0/C18:1-PC-P↓, C18:1/C22:1-PE↓, C18:2/C16:0-PE↓, C18:2/C21:0-PE↓, C18:2/C22:1-PE↓, C20:2/C18:2-PE↓, C20:3/C20:2-PE↓, C20:3/C22:0-PE↓, C20:4/C18:0-PE↓, C20:4/C20:0-PE↓, C16:0/C18:0-PE-P↓, C18:0/C13:0-PE-P↓, C18:0/C17:0-PE-P↓, C18:1/C22:1-PE-P↓, C18:1/C22:1-PE-P↓, C18:1/C22:1-PE-P↓, C18:0/C20:4-PE-P↑, C18:0/C20:5-PE-P↑, C18:0/C22:1-PE-P↓, 20:0/22:6-PE-P↓, C18:0/C16:0-PG↑, C18:1/C18:0-PG↑, C22:6/C20:1-PG↓, C18:2/C18:1-PI↓, C22:6/C16:0-PI↓, C18:0/C21:0-PS↓, C18:1/C20:3-PS↑, C18:1/C22:0-PS↓, C18:1/C24:1-PS↓, 18:2/22:1-PS↓, C20:1/C18:0-PS↓, C20:3/C21:0-PS↓, C22:6/C17:2-PS↑, C22:6/C18:2-PS↓, C18:0/C12:0-SQDG↑	SPMS/PPMS	NAWM	[8]
C20:4/C22:2-DG↓, C18:2/C17:0-PA↓, C20:5/C18:1-PA↓, C18:2/C20:0-PE↑, C20:4/C20:0-PE↑, C20:4/C20:1-PE↓, C22:6/C22:0-PE↓, C18:0/C16:0-PI↓, C18:2/C19:0-PS↓, C20:0-sulfatide↓	PPMS/nCNS and SPMS/nCNS	NAWM	[8]
Total Cer↓, Sph↑, S1P↓, C16:0/C24:0-Cer ratio↑, C18:0/C24:0-Cer ratio↑	MS/nCNS and WM of AD	NAWM	[32]
C18:0-Cer↓, C20:0-Cer↓, C20:0-SM↓, C22:0-SM↓	Ac-MS/nCNS	NAGM	[27]
C20:0-Cer↓, C22:0-Cer↓, C16:0-SM↓	Ac-MS and In-MS/nCNS	NAGM	[27]
C18:0-Cer↓, C20:0-Cer↓, C22:0-SM↓, C24:0-SM↓	Ac-MS and In-MS/nCNS	NAWM	[27]
C18:1(9Z)-lysoPC↑, C18:0-lysoPC↑, C16:0-lysoPI↑, PA↓, PC↑, PI↑	RRMS/ONDs	CSF	[135]
C52:3-TG↓, C58:3-TG↓, C57:4-TG↓, C52:3-TG↓, C61:10-TG↓, C37:2-TG↓, C55:5-TG↓, C57:7-TG↓, C61:8-TG↓, C60:10-TG↓, C62:8-TG↓, C50:1-TG↓, C44:5-TG↓, C59:6-TG↓, C44:4-TG↓, C58:1-TG↓, C56:6-TG↓, C64:10-TG↑, C63:8-TG↑, C59:2-TG↑, C56:4-TG↑, C57:6-TG↑, C32:1-DG↓, C38:7-DG↑, C38:6-DG↑, C32:2-DG↑, C18:3-DG↑, C39:2-DG↑, C42:5-DG↑, C36:6-DG↑, 5beta-cholestane-3alpha↑, 7alpha-diol, 5beta-dihydrotestosterone↑, 22:0 cholesteryl ester↓, cholest-5-en-3alpha-ol↓, 12-methyl-10-oxo- tridecanoic acid↑, N-oleoylethanolamine↑, PE-NMe(O,O-28:0)↑, C21:0-PE↑, C27:1-PC-P↓, C40:3-PS↓, C42:6-PC↑, C25:2-PC↑, C42:0-GluCer↑, C20:0-sulfatide↓, C42:2-C1P↓	RRMS/ONDs	CSF	[136]
C16:0-Cer↑, C24:0-Cer↑, C16:0-HexCer↑	MS/ONDs	CSF	[130]
C16:0-HexCer↑	MS/ONDs	CSF	[155]
24(S)-OHC↑, 24(S)-OHC /27-OHC↑	MS/nCNS	CSF	[48]
24(S)-OHC↓, 27-OHC↓	NZ treated RRMS/untreated RRMS	CSF	[156]
24(S)-OHC↓	MS/ONDs	Serum	[157]
C18:1-lysoPE↓, C18:2-lysoPE↓, C22:4-lysoPE↓, C16:0-lysoPC-P↓, C18:0-lysoPC-P↓, C18:1-lysoPC-P↓, C44:12-PC↓, C20:1-lysoPC↓, C20:0-lysoPC↓, C36:5-PE↓, C35:5-PC↓, C18:1/C18:1-PC↓, C18:0/C18:3-PC↓, tiglylcarnitine↓, 2(R)-HOT↓, GPC(14:0)↓, gamma-linolenic acid↓	PPMS/HS	Plasma	[134]
C18:2-lysoPE↓, C20:0-lysoPC↓, tiglylcarnitine↑	PPMS/RRMS	Plasma	[134]
Gamma-linolenic acid↓, C20:0-lysoPC↓	PPMS/RRMS and PD	Plasma	[134]
C16:0-Cer↑, C24:1-Cer↑, C16:0-GluCer↑, C24:1-GluCer↑, C16:0-LacCer↓	MS/HS	Plasma	[31]
C20:0-HexCer↑, C14:0-SM↓	PPMS/HS	Plasma	[137]
C18:2-LPA↓	PPMS with rapid progression/PPMS with mild progression and SPMS with rapid progression/SPMS with mild progression	Plasma	[137]
LDL-C↓, HDL-C↑	RRMS and progressive MS/HS	Plasma	[158]
C24:0-Cer↓, C16:0-LacCer↓	MS/HS	White blood cells	[31]

All listed SLs or dhCers are based on sphingosine (d18:1) or sphinganine (d18:0) backbones, respectively. Trends are indicated by arrows: down by green and up by red. Abbreviations: Ac-MS—chronic active multiple sclerosis, AD—Alzheimer’s disease, C1P—ceramide 1-phosphate, Cer—ceramide, CSF—cerebrospinal fluid, DG—diacyloglycerol, dhCer—dihydroceramide, GluCer—glucosylceramide, GM—gray matter, GPC—glycerophosphatidylcholine, HDL-C—high-density lipoprotein cholesterol, HexCer—hexosylceramide, HOT—hydroxy-alpha-linolenic acid, HS—healthy subjects, In-MS—chronic inactive multiple sclerosis, LacCer—lactosylceramide, LDL-C—low density lipoprotein cholesterol, LPA—lysophosphatic acid, lysoPC—lysophosphatidylcholine, lysoPC-P—putative lysophosphatidylcholine plasmalogen, lysoPE—lysophosphatidylethanolamine, lysoPI—lysophosphatidylinositol, MS—multiple sclerosis, NAWM—normal appearing white matter, nCNS—normal central nervous system, NZ—natalizumab, OHC—hydroxycholesterol, OND—other neurological disease, PA—phosphatidylanisol, PC—phosphatidylcholine, PC-P—putative phosphatidylcholine plasmalogen, PD—Parkinson’s disease, PE-Nme—phosphtatidylethanolamine-N-methylethanolamine, PE—phosphatidylethanolamine, PE-P—putative phosphatidylethanolamine plasmalogen, PG—phosphatidylglycerol, PI—phosphatidylinositol, PPMS—primary progressive MS, PS—phosphatidylserine, RRMS—relapsing-remitting MS, S1P—sphingosine 1-phosphate, SM—sphingomyelin, Sph—sphingosine, SPMS—secondary progressive MS, SQDG—sulfoquinovosyl diacylglycerol, TG—triacyloglycerol, WM—white matter.

## Data Availability

Not applicable.

## Abbreviations

4-HNE	4-hydroxy-2-nonenal
Ac-MS	chronic active multiple sclerosis
AD	Alzheimer’s disease
ADCC	antibody dependent cellular cytotoxicity
APC	antigen presenting cell
BBB	blood–brain barrier
BDNF	brain-derived neurotrophic factor
C1P	ceramide 1-phosphate
Cer	ceramide
CNS	central nervous system
COX	cyclooxygenase
cPLA_2_α	cytosolic phospholipase A_2_α
CSF	cerebrospinal fluid
DAMP	danger-associated molecular pattern
DC	dendritic cell
DG	diacyloglycerol
dhCer	dihydroceramide
DMF	dimethylfumarate
DMT	disease-modifying therapy
EAE	experimental autoimmune encephalomyelitis
EDSS	expanded disability status scale
EV	extracellular vesicles
FMC	fast migrating cerebroside
Foxp3	forkhead box protein 3
GalCer	galactosylcermide
GL	glycolipid
GluCer	glucosylceramide
GM	gray matter
GPC	glycerophosphatidylcholine
GPI	glycosylphosphatidylinositol
GSL	glycosphingolipids
HDL-C	high-density lipoprotein cholesterol
HexCer	hexosylceramide
HNE	hydroxynonenal
HODE	hydroxy-octadecanoic acid
HOG	human oligoendroglioma
HOT	hydroxy-alpha-linolenic acid
HS	healthy subjects
ICAM-1	intercellular adhesion molecule 1
IFN-β	interferon β
IFN-γ	interferon γ
Ig	immunoglobulin
IL	interleukin
iNKT cells	invariant natural killer T cells
In-MS	chronic inactive multiple sclerosis
I-OND	inflammatory other neurological disease
iTCR	invariant T cell receptor
iTreg	induced regulatory T cell
LacCer	lactosylceramide
LDL-C	low-density lipoprotein cholesterol
LPA	lysophosphatic acid
LPS	lipopolysaccharide
LXR	nuclear liver X receptor
lysoPC	lysophosphatidylcholine
lysoPC-P	putative lysophosphatidylcholine plasmalogen
lysoPE	lysophosphatidylethanolamine
lysoPI	lysophosphatidylinositol
lysoPL	lyso phospholipid
MDA	malondialdehyde
MHC	major histocompatibility complex
MMP	matrix metalloproteinase
MRI	magnetic resonance image
MS	multiple sclerosis
NAWM	normal appearing white matter
nCNS	normal central nervous system
NCXs	sodium calcium exchangers
NFL	neurofilament light chains
NGF	nerve growth factor
NI-OND	non-inflammatory other neurological disease
NKT cells	natural killer T cells
NLR	NOD-like receptors
NO	nitric oxide
Nrf2	nuclear factor erythroid-derived 2-like 2
nTreg	natural regulatory T cell
NZ	natalizumab
OCB	oligoclonal bands
OHC	hydroxycholesterol
OND	other neurological disease
PA	phosphatidylanisol
PAMP	pathogen-associated molecular pattern
PC	phosphatidylcholine
PC-P	putative phosphatidylcholine plasmalogen
PD	Parkinson’s disease
PE	phosphatidylethanolamine
PE-NMe	phosphtatidylethanolamine-N-methylethanolamine
PE-P	putative phosphatidylethanolamine plasmalogen
PG	phosphatidylglycerol
PI	phosphatidylinositol
PL	phospholipid
PPMS	primary progressive multiple sclerosis
PRR	pattern recognition receptor
PS	phosphatidylserine
PUFA	polyunsaturated fatty acid
ROS	reactive oxygen species
RRMS	relapsing-remitting multiple sclerosis
S1P	sphingosine 1-phosphate
S1PR	sphingosine 1-phosphate receptor
SL	sphingolipid
SM	sphingomyelin
Sph	sphingosine
SphK	sphingosine kinase
SPMS	secondary progressive multiple sclerosis
SSPE	subacute sclerosing panenecephalitis
SQDG	sulfoquinovosyl diacylglycerol
SREBPs	sterol regulatory element-binding proteins
TCR	T cell receptor
TG	triacyloglycerol
Th	T helper
TLR	Toll-like receptor
TNF-α	tumor necrosis factor α
Treg	regulatory T cell
VCAM-1	vascular cell adhesion molecule 1
WM	white matter
